# Symptomatic Necrosis With Dual Immune-Checkpoint Inhibition and Radiosurgery for Brain Metastases

**DOI:** 10.1001/jamanetworkopen.2025.4347

**Published:** 2025-04-09

**Authors:** Eugene J. Vaios, Rachel F. Shenker, Peter G. Hendrickson, Zihan Wan, Donna Niedzwiecki, David Carpenter, Warren Floyd, Sebastian F. Winter, Helen A. Shih, Jorg Dietrich, Chunhao Wang, April K. S. Salama, Jeffrey M. Clarke, Karen Allen, Paul Sperduto, Trey Mullikin, John P. Kirkpatrick, Scott R. Floyd, Zachary J. Reitman

**Affiliations:** 1 Department of Radiation Oncology, Duke University Medical Center, Durham, North Carolina; 2 Duke Cancer Institute Biostatistics, Duke University Medical Center, Durham, North Carolina; 3 Department of Biostatistics and Bioinformatics, Duke University Medical Center, Durham, North Carolina; 4 Department of Radiation Oncology, MD Anderson Cancer Center, Houston, Texas; 5 Division of Neuro-Oncology, Department of Neurology, Massachusetts General Hospital, Boston; 6 Department of Radiation Oncology, Massachusetts General Hospital, Boston; 7 Department of Medical Physics, Duke University Medical Center, Durham, North Carolina; 8 Division of Medical Oncology, Department of Medicine, Duke University Medical Center, Durham, North Carolina; 9 Department of Neurosurgery, Duke University Medical Center, Durham, North Carolina; 10 Department of Pathology, Duke University Medical Center, Durham, North Carolina

## Abstract

This cohort study evaluates whether immune-checkpoint inhibition therapy concurrent with radiosurgery is associated with risk of symptomatic radionecrosis among US patients with brain metastases.

## Introduction

Dual immune-checkpoint inhibition (eg, ipilimumab with nivolumab) has significantly improved survival in advanced melanoma and non–small cell lung cancer (NSCLC) and is frequently combined with radiosurgery for the treatment of brain metastases.^1,2,3^ Radionecrosis, a delayed form of brain injury after radiosurgery, is a serious neuro-oncologic challenge with significant patient morbidity and mortality. We investigated whether concurrent treatment with dual immune-checkpoint blockade and radiosurgery is associated with increased risk of symptomatic radionecrosis.

## Methods

This cohort study was approved by the Duke University Health System institutional review board under a waiver of informed consent because it was considered minimal risk given its retrospective nature. This study adheres to STROBE reporting guidelines. Patients with NSCLC or melanoma treated with radiosurgery for brain metastases between January 2014 and August 2022 were identified from a prospective institutional database. Race and ethnicity data are not reported, as they are not radionecrosis risk factors. Systemic therapies and their timing relative to radiosurgery were recorded. Immunotherapy within 4 weeks before or after radiosurgery was considered concurrent, as previously described.^3,4^

Radionecrosis was scored at first imaging evidence (eMethods in Supplement 1). Biopsy-confirmed cases showed no viable cancer. Clinical diagnosis was based on response to steroids or bevacizumab, radiographic evolution, and multidisciplinary tumor board consensus.^3,4,5^ Symptomatic radionecrosis was graded based on the Common Terminology Criteria for Adverse Events.

Control cohorts included patients receiving single (eg, pembrolizumab) or no immune-checkpoint inhibition and were censored at last follow-up or death. Time zero was defined as the date of radiosurgery completion. The Gray method was used to compare the cumulative incidence of symptomatic radionecrosis, accounting for death as a competing risk. Overall survival was evaluated using the Kaplan-Meier method and the log-rank test. Cox univariate and multivariate analyses were performed and estimated hazard ratios (HRs) and 95% CIs were reported. Two-sided *P* < .05 indicated statistical significance. Analyses were performed with SAS, version 9.4 (SAS Institute), and R, version 4.3.1 (R Project for Statistical Computing).

## Results

Overall, 288 patients (134 women [47%] and 154 men [53%]; median [IQR] age, 64 [56-71] years) were included (Table).^3^ At a median follow-up of 58.8 months, 51 patients (18%) developed symptomatic radionecrosis, with pathologic confirmation in 27 (53%). After accounting for other patient and treatment-related factors, only dual immune-checkpoint inhibition was associated with increased risk for symptomatic radionecrosis (HR, 2.4 [95% CI, 1.3-4.7]; *P* = .01). The 24-month cumulative incidence of symptomatic radionecrosis was 21.8% (95% CI, 13.0%-32.1%), 13.5% (95% CI, 8.2%-20.2%), and 13.7% (95% CI, 7.0%-22.7%) following dual, single, and no immune-checkpoint inhibition (Figure, A), respectively. Following concurrent dual and single immune-checkpoint inhibition, 24-month estimates were 25.9% (95% CI, 14.8%-38.5%) and 12.3% (95% CI, 6.0%-21.1%) (Figure, B), respectively. This association was maintained after stratification by histology. Survival was significantly reduced for patients with symptomatic radionecrosis within 1 year of radiosurgery (with radionecrosis: median, 6.9 [95% CI, 6.4-10.7] months; without: 46.0 [95% CI, 35.7-57.6] months; *P* < .001).

**Table.** Baseline Patient and Brain Metastasis Characteristics

Characteristics	Patients, No. (%)		
	Melanoma	NSCLC	Total
**Patient**			
Patients, No.	128	160	288
Age, median (IQR), y	64 (55-71)	64 (56-70)	64 (56-71)
Sex			
Female	54 (42)	80 (50)	134 (47)
Male	74 (58)	80 (50)	154 (53)
Karnofsky performance status			
90-100	95 (75)	103 (64)	198 (69)
70-80	23 (18)	51 (32)	74 (26)
<70	9 (7)	6 (4)	15 (5)
Histology			
Melanoma	128 (100)	0	128 (44)
NSCLC, adenocarcinoma	0	135 (84)	135 (47)
NSCLC, squamous	0	13 (8)	13 (5)
NSCLC, NOS	0	12 (8)	12 (4)
Prior WBRT	9 (7)	6 (4)	15 (5)
Prior chemotherapy	8 (6)	74 (46)	82 (28)
Prior craniotomy	39 (30)	41 (26)	80 (28)
Neurologic deficits at presentation	63 (49)	90 (56)	153 (53)
Prior lines of systemic therapy, median (IQR), No.	1 (0-2)	1 (0-2)	1 (0-2)
Treated brain metastases, median (IQR), No.	3 (2-12)	3.5 (1-6)	3 (1-6)
SRS courses per patient, median (IQR), No.	1 (1-1)	1 (1-1)	1 (1-1)
Immunotherapy exposure			
Dual	67 (52)	15 (10)	82 (28)
Single	47 (37)	82 (51)	129 (45)
None	14 (11)	63 (39)	77 (27)
**Brain metastasis**			
Brain metastases, No.	865	839	1704
Resection cavity	41 (5)	43 (5)	84 (5)
Brain location			
Supratentorial	747 (86)	633 (75.5)	1380 (81)
Infratentorial	80 (9)	169 (20)	249 (15)
Periventricular	30 (4)	28 (3)	58 (3)
Brainstem	7 (1)	9 (1)	16 (1)
Histology			
Melanoma	865 (100)	0	865 (51)
NSCLC, adenocarcinoma	0	732 (87)	732 (43)
NSCLC, squamous	0	63 (8)	63 (4)
NSCLC, NOS	0	44 (5)	44 (2)
Fractionated SRS	416 (48)	260 (31)	676 (40)
Dose for fractionated SRS courses, median (IQR), Gy	27.5 (25-27.5)	25 (25-27.5)	27 (25-27.5)
Fractions for fractionated SRS courses, median (IQR), No.	5 (5-5)	5 (5-5)	5 (5-5)
Single-fraction SRS	448 (52)	573 (68)	1021 (60)
Dose for single-fraction SRS courses, median (IQR), Gy	20 (18-20)	20 (20-20)	20 (19-20)
Planning target volume, median (IQR), cm^3^	0.3 (0.1-1.1)	0.3 (0.1-1.4)	0.3 (0.1-1.2)

Abbreviations: NOS, not otherwise specified; NSCLC, non–small cell lung cancer; SRS, stereotactic radiotherapy; WBRT, whole-brain radiotherapy.

**Figure.**
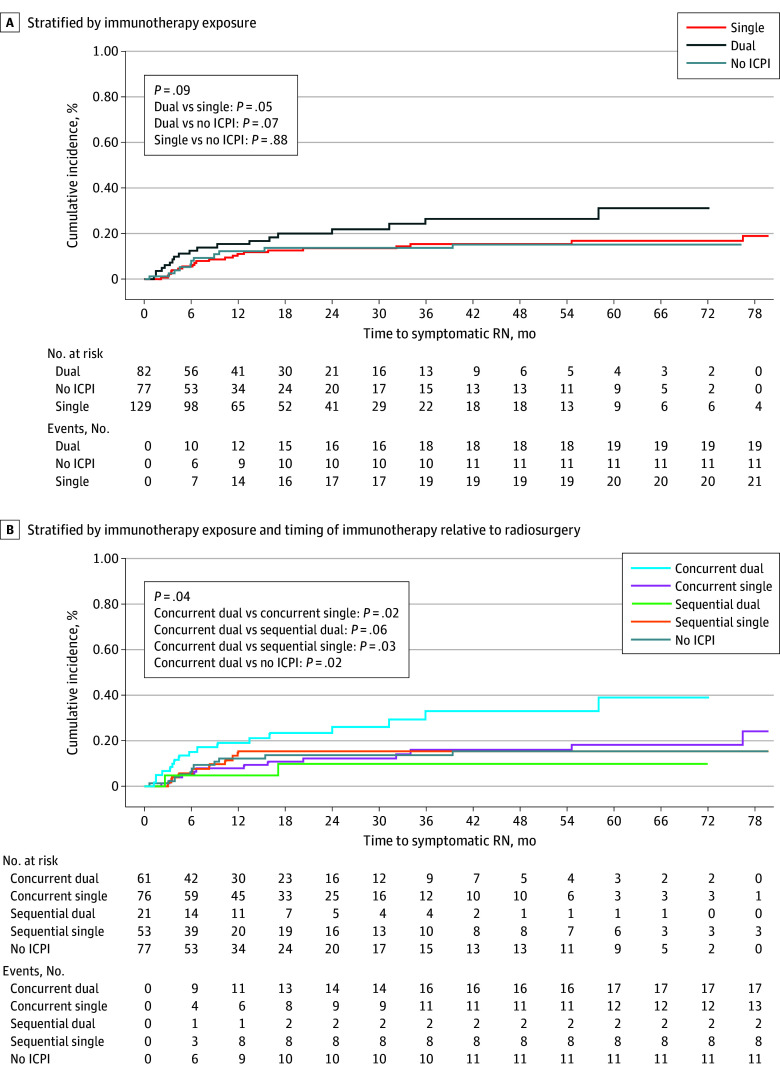
Cumulative Incidence of Symptomatic Radionecrosis (RN) by Immunotherapy Exposure and Timing of Immunotherapy Concurrent indicates immunotherapy exposure within 30 days of radiosurgery; and ICPI, immune-checkpoint inhibition.

## Discussion

In a large cohort of patients with melanoma and NSCLC brain metastases, concurrent dual immune-checkpoint inhibition was significantly associated with increased risk of symptomatic radionecrosis, with potential implications for survival. This finding is consistent with reports of increased symptomatic radionecrosis following concurrent antibody-drug conjugate therapy and radiosurgery.^5^

Despite the increasing use of radiosurgery and brain-penetrating systemic therapies in clinical practice, to our knowledge there are no adequately powered randomized trials to guide the optimal sequencing of treatments. Immunotherapy may prime the tumor microenvironment and amplify radiation-induced immune responses, thereby increasing radionecrosis risk and morbidity.^6^ When clinically feasible, clinicians should consider delaying radiosurgery by 4 weeks or offer fractionated radiosurgery in patients receiving dual-immune checkpoint blockade. Radiosurgery dosing, treatment planning constraints, and the frequency of postradiosurgery surveillance imaging should be reevaluated in patients receiving concurrent treatments.

Study strengths include the large cohort size, controls from a prospective database, and competing risk modeling. The nonrandomized, retrospective nature and pooled analysis of NSCLC and melanoma limit interpretation. Ongoing trials (including NRG-BN013, ABC-X, HYPOGRYPHE) are needed to validate these findings.

## Notes

Supplement 1. **eMethods. **Supplementary Methods

Supplement 2.
Data Sharing Statement
