# Supplementary material for: Symptomatic Necrosis With Dual Immune-Checkpoint Inhibition and Radiosurgery for Brain Metastases
Source: JAMA Netw Open. 2025 Apr 9;8(4):e254347. doi: 10.1001/jamanetworkopen.2025.4347 (PMC11983232; doi:10.1001/jamanetworkopen.2025.4347)
Supplement: Supplement 1. — eMethods. Supplementary Methods [file jamanetwopen-e254347-s001.pdf]

Supplemental Online Content

Vaios EJ, Shenker RF, Hendrickson PG, et al. Symptomatic necrosis with dual immune-checkpoint inhibition and radiosurgery for brain metastases. *JAMA Netw Open.* 2025;8(4):e254347. doi:10.1001/jamanetworkopen.2025.4347

eMethods. Supplementary Methods

This supplemental material has been provided by the authors to give readers additional information about their work.

© 2025 Vaios EJ et al. *JAMA Network Open*.

eMethods. Supplemental Methods

Patients included in the present cohort study were identified from a prospective institutional database, and are previously described[3]. Among patients with NSCLC, 42 patients (26%) were PD-L1 high. EGFR, KRAS, and ALK mutations were detected in 20%, 17%, and 2%, respectively. Among patients with melanoma, BRAF, NRAS, and KIT mutations were detected in 51%, 13%, and 3%, respectively. In the overall cohort, 147 patients (51%) received single fraction radiosurgery. The frequency of single fraction radiosurgery was 48%, 57%, and 46% for patients treated with dual, single, and no immune-checkpoint blockade, respectively. 137 patients (65%) received concurrent immunotherapy plus radiosurgery. Among patients treated with dual or single immune-checkpoint blockade, the frequency of concurrent therapy was 74% and 59%, respectively.

The following criteria were required to qualify for radionecrosis after single or multi-fraction radiosurgery:

1) A pathologic diagnosis based on resection or biopsy revealing radionecrosis without evidence of viable cancer
2) A clinical diagnosis based on response to steroids or bevacizumab (revealing decrease in edema and stability or improvement in contrast enhancing lesions on serial follow up MRIs >3 months) or advanced imaging consistent with radiation necrosis (MR spectroscopy or MR perfusion). Equivocal cases were reviewed at a multi-disciplinary tumor board attended by neurosurgeons, radiation oncologists, medical oncologists, neuro-oncologists, and neuro-radiologists, and a clinical determination of radiation necrosis was made by consensus.

Symptomatic radionecrosis was defined using the Common Terminology Criteria for Adverse Events. Only cases with ≥ grade 2 CNS necrosis were recorded as symptomatic.

© 2025 Vaios EJ et al. *JAMA Network Open*.
