# Supplementary material for: Symptomatic Necrosis With Dual Immune-Checkpoint Inhibition and Radiosurgery for Brain Metastases
Source: JAMA Netw Open. 2025 Apr 9;8(4):e254347. doi: 10.1001/jamanetworkopen.2025.4347 (PMC11983232; doi:10.1001/jamanetworkopen.2025.4347)
Supplement: Supplement 2. — Data Sharing Statement [file jamanetwopen-e254347-s002.pdf]

Data Sharing Statement

Vaios. Symptomatic Necrosis With Dual Immune-Checkpoint Inhibition and Radiosurgery for Brain Metastases. *JAMA Netw Open*. Published April 09, 2025.
doi:10.1001/jamanetworkopen.2025.4347

Data
Data available: Yes
Data types: Deidentified participant data, Participant data with identifiers, Data (not involving human participants), Data dictionary
How to access data: Reasonable data requests for the deidentified outcomes data will be provided upon request to the corresponding author at zjr@duke.edu
When available: With publication

Supporting Documents
Document types: None

Additional Information
Who can access the data: Anyone requesting the data upon reasonable request and data transfer agreement execution.
Types of analyses: For any purpose.
Mechanisms of data availability: With investigator support and a signed data access agreement.
